# Exploring the Gaps in Practical Ethical Guidance for Animal Welfare Considerations of Field Interventions and Innovations Targeting Dogs and Cats

**DOI:** 10.3390/ani8020019

**Published:** 2018-01-27

**Authors:** Louisa Tasker, Susan F. Getty, Joyce R. Briggs, Valerie A.W. Benka

**Affiliations:** 1 Independent Consultant, Hillcrest, Stanton-by-Dale, Derbyshire DE7 4QQ, UK; 2 Alliance for Contraception in Cats & Dogs, 11145 NW Old Cornelius Pass Road, Portland, OR 97231, USA; susan@acc-d.org (S.F.G.); Joyce@acc-d.org (J.R.B.); valerie@acc-d.org (V.A.W.B.)

**Keywords:** domestic dog, domestic cat, animal welfare, ethical review, ethical decision-making, innovation, field intervention, practical guidance

## Abstract

**Simple Summary:**

Large populations of domestic dogs and cats are found living, or in close association with humans. They are often targeted by field interventions or innovations to enhance their welfare or to reduce conflict with communities or wildlife. Ethical review is a cornerstone of responsible engagement that aims to promote animal and human wellbeing. For the review process to be robust, identifying and understanding the ethical dilemmas that may be encountered when working with dogs and cats in field contexts, together with their human communities and in multi-stakeholder partnerships would be advantageous. We explored existing guidance from other disciplines (regulated animal research, veterinary and human clinical trials, and research conducted on wildlife) and identified gaps in ethical frameworks that do not adequately address the specific and practical needs of nongovernmental or intergovernmental organizations, government agencies or independent researchers working with dogs and cats in field contexts. Navigating practical ethical concerns in complex, highly variable field contexts necessitates the development of additional resources that can better inform reliable ethical review processes, and subsequently enhance the humaneness and effectiveness of future interventions and innovations.

**Abstract:**

Domestic dogs (*Canis lupus familiaris*) and cats (*Felis silvestris catus*) are common species targeted by nongovernmental or intergovernmental organizations, veterinarians and government agencies worldwide, for field interventions (e.g., population management, rabies vaccination programs) or innovations (e.g., development of technologies or pharmaceuticals to improve animal welfare). We have a moral responsibility to ensure that the conduct of this work is humane for dogs or cats, and to consider the human communities in which the animals live. Ethical review is widely accepted as being integral to responsible practice, and it is fundamental to good science that underpins innovation. Despite the necessity of field interventions or innovations to advance the welfare of individuals or populations of animals, we found a lack of specific guidance and review processes to help navigate ethical dilemmas surrounding the conduct of such work. This can be detrimental to the wellbeing of animals and their human communities. Here we identify the gaps in existing ethical frameworks (specifically application of Reduction and Refinement principles, challenges of obtaining meaningful informed consent with variations in the quality of human-animal relationships, and limited resources regarding considerations of local stakeholders), and outline the need for additional tools to promote ethical conduct in the field.

## 1. Introduction

Domestic dogs (*Canis lupus familiaris*) and cats (*Felis silvestris catus*) are prevalent and widely distributed [1,2], with global numbers estimated to be 700 and 500 million, respectively [2,3]. They are found in close temporal and spatial association with humans [1,4]. The characteristics and quality of these associations is highly variable and can profoundly impact the welfare of dogs and cats, the human communities where they are found and the wider ecosystem [5,6]. It is not surprising that dogs and cats, regardless of ownership status, are frequently targeted for intervention worldwide, as organizations, veterinarians and government agencies respond to concerns about perceived overpopulation, zoonoses, public health nuisances, animal welfare and impacts on wildlife [7]. These field interventions or innovations may include approaches to manage populations and to control or eradicate disease; they may also involve the research and development of new technologies and pharmaceuticals (e.g., nonsurgical fertility control and marking technologies [8]). Their common purpose is to enhance animal wellbeing and/or reduce risks to humans, and they are increasingly advanced under the One Health concept [9], which necessitates multi-sectorial involvement. Over their lifetime, these types of field projects experience a suite of highly variable and complex circumstances and inevitably present ethical challenges. Individuals and organizations undertaking field work have a moral obligation to ensure that projects prioritize the wellbeing of both target animals and broader communities by undertaking ethical review. 

Ethical review is widely accepted as being integral to promoting humane, responsible practice and science. It provides a formal process to promote decision-making and functions as an evaluation tool to examine the ethical appropriateness of projects [10]. Its most important function is arguably a protective one [11], to serve as a mechanism to anticipate and then obviate or mitigate potential harms [12]. To be effective and robust, the process should include systematic consideration of a given set of circumstances [10], by a suitably qualified and competent ethical review body, consisting of diverse membership that can adequately consider the interests of all stakeholders (e.g., animal welfare, human wellbeing, research/study design and lay members). The process should reflect on the ethical challenges likely to be faced and provide a contextual framework within which challenges can be viewed. It is our concern that no appropriate ethical guidance or recommendations for formal review processes exist for conducting studies or projects with dogs and cats in a field context when the interventions and innovations are designed to help those animals specifically, or others of their species [13].

For the purposes of our work, we refer to “innovation” in broad terms, relating to the advancement of new knowledge through the development of ideas, methods, products and practices; here we apply it to field contexts in finding an answer to an important problem, that addresses an important need (scientific necessity), which may also include translation of knowledge across species and contexts. Scientific necessity requires considerations of the quality of scientific output of animal studies [14]. “Intervention” refers to actions or processes that aim to improve animal welfare more broadly; they may overlap with innovation when the advancement or application of methods or practices involves scaling up or expanding geographical scope. Similarly, the necessity of intervention requires an attempt to quantify (where possible) the welfare need and rationale for intervening [3,15]; it too, is fundamentally concerned with finding a resolution to an important problem. We consider field contexts to be those outside the confines of a laboratory. They may include conditions that are designed to mimic naturally occurring environments in target animal populations (cats or dogs); they may also refer to work undertaken in populations where those animals are managed or kept by humans (e.g., shelters, veterinary practices, owned animals, roaming animals or those cared for by communities), and span international geographical contexts (e.g., low-, middle- or high-income countries).

Approval to conduct regulated animal studies, veterinary practice-based research, field studies with wildlife (reviewed in Table 1) and research involving vulnerable human populations (Table 2), requires justification for the necessity of such research, including weighting the balance of harms (to research subjects) against the suitability of the science and intended benefits to human welfare or other animals. In some of these contexts, there are legal requirements and professional recommendations for review procedures (via local Institutional or Animal Welfare and Ethical Review Bodies). Given that many organizations that implement field interventions—the focus of this article, operate outside of the academic or regulatory arena, the lack of ethical guidance poses a particular challenge [16]. This may be further compounded when field work is undertaken in countries where animal welfare or veterinary capacity is limited, and the safeguarding practice of ethical review is also absent or inadequate.

## 2. Methods

To explore the appropriateness of existing approaches and identify gaps in guidance and the literature, the Alliance for Contraception in Cats and Dogs (ACC&D) conducted a rapid review and convened twenty-four expert academics and field practitioners [17] at a Think Tank entitled ‘Ethical Decision-Making in Innovation for Animal Welfare’ [18]. The initial review process was completed over a period of weeks in advance of the Think Tank (described below). It involved topic selection by ACC&D staff and volunteers, as well as invited Think Tank attendees. Topic categories included animals in research, veterinary medicine (comprising clinical trials/novel therapeutic interventions) and the use of human participants in clinical trials. Relevant literature according to topic was identified through personal experience, Google Scholar, contact via professional networks and Think Tank participants; additional snowballing of literature from the reference lists also produced relevant literature for review. Alliance for Contraception in Cats & Dogs staff members, divided up, read and summarized peer-reviewed and grey literature, and scored 1–5, with 1 being the least relevant to the selected topics. One staff member then extracted the data to produce a narrative summary [13]. 

The Think Tank was convened over two days; it utilized an Appreciative Inquiry approach [19] with a single facilitator to engage the participants’ multiple perspectives to address the deficits identified above. Below, we provide a commentary of the practical ethical considerations, identified with the assistance of the Think Tank [17], which may be relevant to scientific, veterinary and animal welfare communities seeking to advance animal welfare through intervention or innovation targeting dogs and cats in field contexts.

## 3. Results and Discussion

Drawing from the literature review, Table 1 summarizes ethical guidance on the use of animals for research conducted in a range of settings (regulated experiments on animals, veterinary clinical studies, novel veterinary interventions and research conducted on free-living wildlife). Table 2, for comparison, gives a brief overview of guidance for conducting human clinical trials with a focus on international settings. Three key themes that became evident in the review of the literature during the Think Tank and subsequent research, which have relevance to ethical decision-making and ethical review for field-based interventions and innovations are discussed below.

### 3.1. Applications and Considerations from Existing Ethical Guidance for Dogs and Cats in Field Contexts

#### 3.1.1. Application of 2 Rs, Reduction and Refinement

Scientific or therapeutic necessity, providing answers to an important problem, is the justification for undertaking research on animals, and may be similarly relevant for justifying an intervention or innovation. A common approach adopted for dealing with the ethical dilemma of using animals in research is utilitarian, which requires the weighting of harms (to animals) vs. benefits (to humans or other animals) [51]. For interventions, both the necessity (welfare need) and the appropriateness of design (whether the intervention has a reasonable chance of meeting stated goals) should be balanced against potential harms to animals. In promoting the reduction of harms to animals, Replacement, one of the 3Rs principles (Replacement, Reduction, Refinement [22]) that underpins regulated animal use, does not apply for interventions, or field studies undertaken to meet the requirements for safety and efficacy testing during product development [52], as they are conducted on the target species. The second principle of humane science, Reduction (methods used to reduce the number of animals per study [22]), should be applied to innovation-focused research where the risks of adverse effects are poorly understood.

In the laboratory, animals have the potential to experience pain, suffering, distress or lasting harm [23]. The third principle, Refinement, seeks to address these by promoting approaches to avoid or minimize harm, and proactively enhance welfare over the animal’s lifetime through to death [53]. This necessitates refining practices directly related to the conduct of procedures (e.g., adverse events) and those indirectly associated with the inclusion of animals in research (e.g., housing and husbandry) [54]. Although animals targeted for intervention may be subjected to similar human-mediated actions (e.g., capture, handling, restraint, surgery and temporary housing), the harms these actions cause may not be identified or quantified, nor suitable methods employed to obviate or mitigate such actions [55].

A systematic framework for mapping welfare considerations is recommended for innovations or interventions that could impact animal welfare through capture, handling, restraint, surgery, dosing or other adverse events. This practical approach is not currently available for animals in field conditions, but it is advocated to reduce or prevent severe suffering in laboratory-housed animals [56]. The recommended ‘Road Map’ of practical steps—audit, evaluation, define and overcome obstacles associated with research conducted on animals [56] aids compliance with the ‘spirit’ of UK legislation [57]. All sources of suffering (direct and contingent) are identified over the lifetime of the animal. This entails breaking down lifetime experience to component steps, such as the animal’s source (conditions under which the animal was bred, reared and cared for), transport (e.g., mode, duration, conditions), methods of marking/identification, housing, husbandry, capture, handling, restraint, adverse effects related to procedures, and killing. For each step the aim is to analyze the potential or actual suffering and apply or evaluate refinements [56]—RSPCA, LASA, LAVA, IAT resource packs [57,58,59,60], provide detailed practical guidance for ethical review bodies on the ‘Road Map’ approach. Given the potentially unique and significant vulnerabilities of dogs and cats in field contexts (e.g., poor nutrition, poor health and limited socialization with humans), careful consideration should be given to whether individuals can cope with the ensuing short-term harms of intervention, even if the intention is that the individual may benefit in the longer-term. These factors could be balanced through the weighting of harms to benefits when animals are included in field interventions or innovations and should be considered in light of the consequences of not intervening at all (e.g., the risk of inhumane culling for population or disease control [3,15].

#### 3.1.2. Balancing Risk/Benefit and Informed Consent

A similar system of balancing risks and benefits is fundamental to ethical oversight of human clinical trials, but with a greater emphasis placed on the protection of the participant, which takes priority over scientific necessity [40,41,42]. Approval is based upon a different set of ethical principles such as non-maleficence, beneficence, protection, autonomy and social justice; it requires participants to give informed consent, with an option for voluntary withdrawal [43]. Similarly, the decision-making surrounding the use of novel therapeutic interventions in veterinary practice, balances the principles of non-maleficence (do no harm), against beneficence (do good); the animal must have a reasonable chance of benefiting, and this potential benefit must outweigh the anticipated risks associated with treatment [31,35].

Given that research based in veterinary practice or veterinary clinical trials are undertaken on owned animals, it is imperative that the owner or keeper of the animal is included in the decision-making process [28,29,30,31,32,33,34,35]. A process of informed consent must be followed that ensures consent is meaningful—based on sound understanding of the purpose of the research (and comprehension of benefits and risks to the animal), and that it is voluntary, freely given (without coercion or undue incentive) and documented [31,32,35]. This requires careful communication to the owner, and the consent dialogue may need to take into consideration the perspectives and motivations of the owner (which may be more broadly impacted by the human-animal bond), the power differential between owners and researchers [28], and social, cultural or educational norms [61]. The inclusion of an owned animal for clinical trial or novel therapeutic intervention should also identify potential harms (including distress) to the owner (e.g., related to non-therapeutic effects or decisions surrounding euthanasia for humane endpoints) [31,35]. Nonetheless, with novel therapeutic interventions, the lack of prior safety and efficacy information makes presenting an account of the anticipated risks/benefits, in a consent dialogue inherently difficult [31]. Furthermore, with veterinary clinical trials, there may be an additional concern, that of therapeutic misconception; owners misunderstand that the research outcomes are not the same as treatment outcomes (e.g., animals in different treatment or control groups may not benefit from inclusion in the study, and the researchers may be blinded to treatment groups). This effect has been widely recognized in the conduct of human clinical trials [62].

#### 3.1.3. Community and Stakeholders

Large populations of dogs and cats worldwide may not have a single identifiable owner [7]. They may experience fluctuating levels of basic care intentionally provided by a community [63] or they may live independently and actively avoid humans [1,4]. Interventions and innovations may deliberately target animals that span these different states (based upon identified welfare or public health need, e.g., catch-neuter-release, trap-neuter-return, rabies vaccination campaigns, and nonsurgical fertility control). For these animals, the human communities where they live require consideration. Communities can be directly impacted through the concerns they hold about how the animals are faring in the environment [64], or they may be seriously affected by public health and wider nuisance concerns [5,6]. Where animals are far less dependent on the provision of human care, as with research conducted on wildlife, the effect of any intervention or innovation on the animals’ ability to interact with the environment that may be detrimental to their welfare should be obviated or mitigated. Examples include increasing instability in social groups; impacting their survival through altering access to resources; or increasing conflict with human communities, other animals such as livestock or conservation efforts where dogs or cats impact wildlife [3,15]. Nevertheless, local stakeholders (e.g., human community, veterinarians, animal caretakers) may have different ethical perspectives when it comes to animals (e.g., contractarian, utilitarian, relational, animal rights and respect for nature), which guide decision-making [65].

To help consolidate differences in ethical values, frameworks and standards of care for human participants in clinical trials undertaken in international contexts, mandated reviews should include processes at local level [45,46]. Local ethical review boards may be better equipped to understand the complexities of the conditions *in situ* [45,46,47,48]. Their inclusion in the review process may help to navigate the interplay between wider stakeholders (e.g., funding or research agencies), target animals, and associated communities. Submitting projects for independent review to appropriate ethical bodies was mandated or strongly recommended (Table 1) as a safeguarding approach for animal welfare, though there are limitations in a process that relies on one-off review (performed prospectively). Ethical oversight is increasingly required throughout projects to fully quantify their impact on animals and review the quality of scientific outcomes [27].

## 4. Conclusions

This commentary is a first exploration of the appropriateness of existing guidance and regulatory oversight for ethical decision-making in interventions or innovations targeting dogs and cats in the field. The literature review process was rapid, and there is geographical bias in the publications consulted (US and European focus and authorship), particularly on ethics of animal (versus human clinical) research, due to what is available in the peer-reviewed literature. However, the early engagement of multidisciplinary experts with knowledge in the field meant the review process focused on literature that was practical and relevant to the scope of our work. The Think Tank also helped hone in on pertinent issues in a difficult and complicated ethical landscape. The review, together with insights from the Think Tank has emphasized the inherent difficulties in navigating these complex, highly variable field contexts. This process has sparked initiatives to develop an interactive tool to promote practical ethical decision-making in the field, with relevance to veterinarians, animal welfare organizations, government agencies, ethical review boards and even project funders. 

The ethical principles applied in current guidance alone do not fully provide a systematic or suitable framework to support decision-making, that promotes humane and responsible practices more widely for animals and the human communities where they live. For those of us working in field contexts, limited guidance for ethical decision-making and the absence of ethical review, may prevent us from adequately identifying, avoiding and mitigating potential and actual harms to animals and humans during the lifetime of the intervention or innovation. Furthermore, journals increasingly require a formal process of ethical review to be undertaken, which if not performed will prevent dissemination of findings from these important projects through peer-reviewed publication.

These types of projects are likely to benefit from additional support and ethical oversight, which in turn requires a better understanding of the ethical issues practitioners, researchers and ethicists have faced when working in the field. Given these difficulties, the current process of formal ethical review, where mandated or available may be inadequate. It is unlikely to consider different ethical frameworks other than utilitarianism that influence decision-making by diverse local stakeholders related to animal welfare, and it may not reflect the interests of human communities where the animals are found, and on whom they may be dependent. Formal ethical review processes may be more robust if they include broader representation and incorporate local stakeholders—although capacity for ethical review may need to be built at local level if there are no suitable existing review bodies, and it may require additional time for review processes to be completed. When organizations are not partnered with academic institutions, where a principle investigator takes responsibility for submitting projects for ethical review, an equivalent competent person will need to be identified to ensure the process of ethical review is followed. This may be the project lead, or individuals carrying out monitoring, evaluation or impact assessment for innovations or interventions.

The ethical requirements of field projects will change over their lifetime, necessitating prospective and intermediate oversight, and retrospective review. Reflective practices are important for capturing learnings to inform the ethical conduct of current and future projects. This knowledge can be consolidated into more appropriate ethical frameworks and shared to guide decision-making by those who work to promote animal welfare in field contexts.

## Figures and Tables

**Table 1:** Brief overview of ethical guidance for conducting research on animals.

Population	Ethical Framework, Ethical Review and Additional Considerations
**Animals housed in laboratories:** Includes research and development of veterinary pharmaceuticals or technologies.	**Scientific necessity/Justification of use:** To benefit human health/welfare—translational research across species. To benefit animals—research is conducted in the target species. The individual often does not benefit.
	**Harm-benefit analysis (HBA):** Weighting of consequences (direct and contingent) for the animals vs. weight of benefit (scientific necessity) [20], which requires demonstration of the scientific rationale [21].
	**Application of the 3Rs principles:** Replacement, Reduction, Refinement [22], impacts the weighting of harms to animals.
	**Ethical review:** Often formal and legally required in some countries [23,24]. Animals may be afforded legal protection [23,24,25]. Review is prospective, conducted before the start of research; predict harms likely to be experienced by the animal. In addition, in Europe, retrospective review is conducted at the end of research, which aims to quantify actual harms experienced by the animal [26,27].
**Veterinary field-based research:** Veterinary clinical trials —includes observational and interventional; undertaken to meet the legal requirements for safety and efficacy testing under field conditions. Novel veterinary therapeutic interventions (e.g., unlicensed pharmaceuticals for target species, medical devices, implants, or surgical techniques) on individuals in veterinary practices.	**Scientific necessity/Justification of use:** To benefit animals—research is conducted in the target species; therapeutic application to other animals. Individual animal may also benefit. Individuals in placebo/controls may not benefit or may experience harm due to sham procedures or lack of therapeutic intervention.
	**HBA:** Weighting of consequences (direct and contingent) for the animals (includes lack of therapeutic effect) vs. weight of benefit (scientific or therapeutic necessity) to target population and/or the individual [28,29,30,31]. Requires demonstration of scientific rationale [28,29,30,31,32]. Note, the lack of safety or efficacy data in target species makes it difficult to weigh benefits and consequences to the individual [29,31,32,33].
	**Application of 2Rs principles** [30,31,32]: Replacement does not apply. Reduction may apply in some instances where there is uncertainty over the benefits to the animal. Refinement should include humane endpoints, given the difficulties with predicting adverse events [31,32].
	**Ethical review:** Variation in review processes depending on necessity; they may be recommended or legally required; prospective and increasingly retrospective [28,29,30,31,32,33].
	**Additional considerations apply:** Animals are owned and afforded legal protection. There are obligations of, and to the owner relating to informed consent (understand risk vs. benefit for the animal; therapeutic misconception; voluntary withdrawal) [28,29,30,31,32,33]. Harm or distress may be experienced by the owner or keeper [34,35]. The complexities of human-animal relationship, the veterinary-client relationship and who is acting in the best interests of the animal may impact decision-making. Veterinarians should be mindful of conflicts of interest (e.g., acting in research capacity, biasing study-related needs vs. professional capacity, prioritizing the individual’s welfare—conflicts with non-malfeasance [31]). Strong recommendations not to undertake novel therapeutic interventions on stray or feral animals (e.g., catch/trap-neuter-release, sheltered; where an owner cannot withhold consent) unless in exceptional circumstances; acting in the best interests of the animal and necessitates external ethical oversight [31].
**Wild animals in field contexts:** Research conducted on free-living wildlife—includes unobtrusive, observational, interventional or invasive studies.	**Scientific necessity/Justification of use:** To benefit the animal population or species. To benefit the wider ecosystem, including humans.
	**HBA:** Weight of consequences (direct and contingent) for the animals vs. weight of benefit (scientific necessity) [34,36,37].
	**Application of 2Rs principles** [37,38,39]: Replacement does not apply.
	**Additional considerations apply:** Deliberate or inadvertent manipulations of the environment or the animals’ behaviour, which may impact their survival by creating disturbances: - Within-species interactions (e.g., social groups, reproductive behaviour, mother-infant interactions); - Between-species interactions (e.g., predator-prey, including hunting by humans—for consumption, trade; impact on human livelihoods); - to species-habitat interactions (e.g., access to food/water/sleeping sites) [34,36,37,38,39].
	**Ethical review:** Recommendations for prospective ethical review [34,37,38]. Legal obligations to animals may apply under certain circumstances [38,39].

**Table 2:** Brief overview of ethical guidance for conducting clinical trials on human participants.

Population	Ethical Framework, Ethical Review and Additional Considerations
**Humans in field contexts:** Clinical trials conducted on human participants with a focus on international settings.	**Scientific necessity/Justification of use:** To benefit human welfare—the individual and/or the population.
	**Harm-benefit analysis (HBA):** Anticipated benefits for the individual or society must outweigh the risks to the individual [40,41,42], which requires demonstration of the scientific quality of the study [41,42,43,44].Strict legislative, regulatory and ethical guidance oversee research conduct [41,42].
	**Ethical principles:** Include—autonomy, protection, non-maleficence, beneficence and justice for human participants [43].
	**Ethical review:** Formal and legally required, and includes local, in-country ethical review by the relevant bodies [45,46]. There are notable variations in the capacity [45] and practice of research ethics [44], which would benefit from a better understanding of social, economic and political contexts in host countries [45,46,47,48].
	**Additional considerations apply:** The conflicts in ethics [49] and standards of human care between countries [47,48]. Identification of risks and benefits to the participant must be appropriately communicated [43]. Researchers must obtain voluntary, informed consent from participants; participants also have the right to voluntarily withdraw from the study [43]. Local variations in factors (e.g., social and educational norms) that impact the researcher’s ability to obtain meaningful, informed consent [40,41,47]. Recommendations for ensuring vulnerable and disadvantaged populations benefit from inclusion into the research (e.g., receive wider improvements in healthcare and social justice [40,47,48]). Community engagement and acceptability is recognized as critical to the success of human clinical trials [48,50].
